# Supplementary material for: The impact of COVID-19 on non-communicable disease patients in sub-Saharan African countries: A systematic review
Source: PLoS One. 2024 Jun 21;19(6):e0293376. doi: 10.1371/journal.pone.0293376 (PMC11192341; doi:10.1371/journal.pone.0293376)
Supplement: S1 Table — (DOCX) [file pone.0293376.s001.docx]

**Appendix . Summary of quality assessments using JBI appraisal checklist.**

| S. no. | Author  Year | Study focus | Items on Joanna Briggs Institute | | | | | | | | Raw score and % | Risk |
| --- | --- | --- | --- | --- | --- | --- | --- | --- | --- | --- | --- | --- |
|  |  |  | **Q1** | **Q2** | **Q3** | **Q4** | **Q5** | **Q6** | **Q7** | **Q8** |  |  |
|  | (Abate et al., 2022)  Ethiopia | Adherence to physical exercise recommendations among type 2 diabetes patients during the COVID-19 pandemic | 1 | 1 | 1 | 1 | 1 | 0 | 1 | 1 | 7/8= 87.5 | low risk of Bias |
|  | (Abdisa et al., 2022). | Factors associated with poor medication adherence during COVID-19 pandemic among hypertensive patients visiting public hospitals | 1 | 1 | 1 | 1 | 1 | 0 | 1 | 1 | 7/8= 87.5 | low risk of Bias |
|  | (Addis et al., 2021) | Psychological impact of COVID-19 pandemic on chronic disease patients | 1 | 1 | 1 | 1 | 0 | 0 | 1 | 1 | 6/8= 75 | low risk of Bias |
|  | (Ajele et al., 2022b) | Relationship between depression and psychological well‑being among persons with diabetes during COVID‑19 pandemic: diabetes distress as a mediator | 1 | 1 | 1 | 1 | 0 | 0 | 1 | 0 | 5/8= 62.5 | Moderate risk of Bias |
|  | (Andualem et al., 2020) | Adherence to Lifestyle Modifications and Associated Factors Among Adult Hypertensive Patients | 0 | 1 | 1 | 1 | 1 | 0 | 1 | 1 | 6/8= 75 | low risk of Bias |
|  | (Awel et al., 2022) | Impact of COVID-19 on Health Seeking Behavior of Patients with Chronic Disease | 1 | 1 | 1 | 1 | 1 | 0 | 1 | 1 | 7/8= 87.5 | low risk of Bias |
|  | (Ayalew et al., 2022) | Quality of life among patients with chronic non-communicable diseases during COVID-19 pandemic | 1 | 1 | 1 | 1 | 1 | 1 | 1 | 1 | 8/8= 100 | low risk of Bias |
|  | (Ayele et al., 2022a) | Effect of COVID-19 pandemic on missed medical appointment among adults with chronic disease conditions | 0 | 1 | 1 | 1 | 0 | 0 | 1 | 1 | 5/8= 62.5 | Moderate risk of Bias |
|  | (Ayele et al., 2022b) | The effect of COVID-19 on poor treatment control among ambulatory Hypertensive and/ or Diabetic patients | 0 | 1 | 1 | 1 | 0 | 0 | 1 | 1 | 5/8= 62.5 | Moderate risk of Bias |
|  | (Belete et al., 2022) | Prevalence of depression and associated factors among adult cancer patients receiving chemotherapy during the era of COVID-19 | 1 | 1 | 1 | 1 | 1 | 0 | 1 | 1 | 7/8= 87.5 | low risk of Bias |
|  | (Ofori et al., 2021) | Psychological impact of COVID-19 on diabetes mellitus patients | 0 | 1 | 1 | 1 | 1 | 0 | 1 | 1 | 6/8= 75 | low risk of Bias |
|  | (Fentaw et al., 2022) | Blood pressure control status of patients with hypertension on treatment | 1 | 1 | 1 | 1 | 1 | 0 | 1 | 1 | 7/8= 87.5 | low risk of Bias |
|  | (Gebeyehu et al., 2022) | Dietary knowledge and practice and its associated factors among type 2 diabetes patients on follow-up | 1 | 1 | 1 | 1 | 1 | 0 | 1 | 1 | 7/8= 87.5 | low risk of Bias |
|  | (Girma et al., 2021) | Covid-19 Pandemic-Related Stress and Coping Strategies Among Adults with Chronic Disease | 1 | 1 | 1 | 1 | 1 | 0 | 1 | 1 | 7/8= 87.5 | low risk of Bias |
|  | (Joseph et al., 2022) | Patient’s Perspective on the Impact of COVID-19 on Cancer Treatment in Nigeria | 1 | 1 | 1 | 1 | 1 | 1 | 1 | 1 | 8/8= 100 | low risk of Bias |
|  | (Kiarie et al., 2022) | The COVID-19 pandemic and disruptions to essential health services | 0 | 1 | 1 | 1 | 1 | 0 | 1 | 1 | 6/8= 75 | low risk of Bias |
|  | (Maphisa and Mosarwane, 2022) | Changes in retrospectively recalled alcohol use pre, during and post alcohol sales prohibition during COVID pandemic | 0 | 1 | 1 | 1 | 0 | 0 | 1 | 1 | 5/8= 62.5 | Moderate risk of Bias |
|  | (Mekonnen et al., 2022) | Impact of COVID-19 on non-communicable disease management services at selected government health centers | 0 | 1 | 1 | 1 | 0 | 0 | 1 | 1 | 5/8= 62.5 | Moderate risk of Bias |
|  | (Mekonnen et al., 2021) | Determinants of Dietary Adherence Among Type 2 Diabetes Patients Aimed COVID-19 | 1 | 1 | 1 | 1 | 1 | 0 | 1 | 1 | 7/8= 87.5 | low risk of Bias |
|  | (Musinguzi et al., 2021) | The Early Impact of COVID-19 on a Cardiovascular Disease Prevention Program | 1 | 1 | 1 | 1 | na | na | 1 | na | 5/5= 100 | low risk of Bias |
|  | (Nshimyiryo et al., 2021) | Barriers and coping mechanisms to accessing healthcare during the COVID-19 lockdown among patients with chronic diseases | 1 | 1 | 1 | 1 | 0 | 0 | 1 | 1 | 6/8= 75 | low risk of Bias |
|  | (Shimels et al., 2021) | Magnitude and associated factors of poor medication adherence among diabetic and hypertensive patients visiting public health facilities in Ethiopia during the COVID-19 pandemic | 1 | 1 | 1 | 1 | 1 | 0 | 1 | 1 | 7/8= 87.5 | low risk of Bias |
|  | (Umar et al., 2022) | COVID-19 and access to cancer care in Kenya: patient perspective | 1 | 1 | 1 | 1 | 1 | 0 | 1 | 1 | 7/8= 87.5 | low risk of Bias |
|  | (Umutoniwase et al., 2022) | Food insecurity and level of depression among patients with chronic diseases, and associated factors during the COVID-19 lockdown | 1 | 1 | 1 | 1 | 1 | 0 | 1 | 1 | 7/8= 87.5 | low risk of Bias |
|  | (Yasin et al., 2022) | COVID-19-related anxiety and knowledge toward its preventive measures among patients with chronic medical illness on follow-up in public hospitals | 1 | 1 | 1 | 1 | 1 | 0 | 1 | 1 | 7/8= 87.5 | low risk of Bias |
|  | (Azzouzi et al., 2022) | The Impact of the COVID-19 Pandemic on Healthy Lifestyle Behaviors and Perceived Mental and Physical Health of People Living with Non-Communicable Diseases: | 1 | 1 | 1 | 1 | 1 | 0 | 1 | 1 | 7/8= 87.5 | low risk of Bias |
|  | (Beyene, 2022) | Routine care of patients with chronic non-communicable diseases | 1 | 1 | 1 | 1 | 1 | 0 | 1 | 1 | 7/8= 87.5 | low risk of Bias |
|  | (Irusen et al., 2021) | COVID-19 Related Anxiety | 1 | 1 | 1 | 1 | 1 | 0 | 1 | 1 | 7/8= 87.5 | low risk of Bias |
|  | (Hiko et al., 2022) | Follow-up conditions of care | 1 | 1 | 1 | 1 | 1 | 0 | 1 | 1 | 7/8= 87.5 | low risk of Bias |
|  | (Mujuru et al., 2023) | The impact of lockdown measures | 1 | 1 | 1 | 1 | 0 | 0 | 0 | 1 | 5/8= 62.5 | Moderate risk of Bias |

**JBI Criteria to be scored:** Q1. Were the criteria for inclusion in the sample clearly defined?, Q2. Were the study subjects and the setting described in detail?, Q3.. Was the exposure measured in a valid and reliable way?, Q4. Were objective, standard criteria used for measurement of the condition?, Q5. Were confounding factors identified?, Q6. Were strategies to deal with confounding factors stated?, Q7. Were the outcomes measured in a valid and reliable way?, Q8. Was appropriate statistical analysis used? 1 = Yes, 0 = No, and U = Unclear.

**Abbreviations:** 1 = Yes; 0 = No; U = Unclear; NA = Not Applicable; JBI: Joanna Briggs Institute

**Criteria used to rank the risk of bias**i) ≤49% = high risk of Bias
ii) 50% and 69% = Moderate risk of Bias

iii) Above 70% = low risk of Bias

Moderate risk of Bias= 6

low risk of Bias= 24
