# Supplementary material for: The impact of COVID-19 on non-communicable disease patients in sub-Saharan African countries: A systematic review
Source: PLoS One. 2024 Jun 21;19(6):e0293376. doi: 10.1371/journal.pone.0293376 (PMC11192341; doi:10.1371/journal.pone.0293376)
Supplement: S1 File — (DOCX) [file pone.0293376.s003.docx]

## Review protocol

|  | Title | The Impact of COVID-19 on Non-Communicable Disease Patients in Sub-Saharan African Countries: A Systematic Review | |
| --- | --- | --- | --- |
|  | Background: | Non-communicable diseases (NCDs), also known as chronic diseases, are long-term illnesses that cannot be transmitted from person to person. These diseases, which include conditions such as diabetes, cancer, and heart disease, are responsible for the majority of global fatalities, accounting for 71% of all deaths worldwide and killing 41 million people annually. The COVID-19 pandemic has had a significant impact on NCD patients in sub-Saharan Africa, where NCDs are a major public health challenge. However, there is limited information on the specific effects of the pandemic on NCD patients in this region. | |
|  | Review purpose | The main aim of this systematic review is to examine the existing literature conducted on the impact of COVID-19 among non-communicable disease patients in sub-Saharan African countries. Specifically, this review will focus on the effects of the pandemic on access to care, mental health outcomes, and risk and protective factors related to NCDs in the region. This analysis will investigate the connection between COVID-19 and other factors. It will also examine how noncommunicable diseases may have contributed to the pandemic's severity and the effect of preventative measures implemented in response to the outbreak. Lastly, this review will provide insight into the overall efficacy of the interventions and make policy recommendations to improve health outcomes for those in the region with NCDs. | |
|  | Review questions | "What is the impact of COVID-19 on access to care, mental health, and risk and protective factors related to non-communicable diseases (NCDs) among patients in sub-Saharan African countries?". | |
|  | Inclusion criteria | Studies will be eligible for inclusion in the review if they:   - Are published in English - Report on the impact of COVID-19 on NCD patients in sub-Saharan African countries - Focus on one or more of the review objectives (access to care, health outcomes, other factors related to NCDs) - Are primary research studies | |
|  | Exclusion criteria: | Studies will be excluded from the review if they:   - Are not published in English - Do not report on the impact of COVID-19 on NCD patients in sub-Saharan African countries - Do not focus on one or more of the review objectives (access to care, health outcomes, other factors related to NCDs) - Are not primary research studies (e.g. review articles, editorials) | |
|  | Outcome measures | •    Access to care for NCDs: The study aims to explore the ways in which the COVID-19 pandemic has disrupted access to care for NCDs in sub-Saharan African countries, including factors such as reduced capacity for non-emergency services, delays in diagnosis and treatment, and decreased adherence to treatment regimens. •    Mental health outcomes for NCD patients: The study aims to examine the impact of the COVID-19 pandemic on the mental health of NCD patients in sub-Saharan African countries, including factors such as anxiety, depression, and stress. •    Risk and protective factors related to NCDs: The study aims to explore the ways in which the COVID-19 pandemic has impacted risk and protective factors related to NCDs in sub-Saharan African countries, such as substance use, exercise, and lifestyle changes. |  |
|  | Expected outcome | The results of this review will provide a comprehensive overview of the existing literature on the impact of COVID-19 on NCD patients in sub-Saharan African countries.  The review will highlight any inconsistencies or gaps in the literature and will inform future research in this area.  The review will inform efforts to address the impact of COVID-19 on NCD patients in sub-Saharan African countries and will contribute to the global understanding of the pandemic's impact on healthcare systems and patient outcomes. | |
|  | Search strategy | The search will be done in two phases. In the first phase, database searching without a time setting will be conducted on selected databases, including MEDLINE, EMBASE, and CINAHL. Furthermore, manual searching from references and unpublished works of literature will be explored in the gray literature and Google Scholar. | |
|  | Study selection | First, the title and abstract of the research identified through the search strategy will be examined. Then, if they are considered relevant, the full text will be examined by exporting into Covidence. Once the full texts are exported into Covidence, they will be assessed for eligibility by two independent reviewers according to the pre-defined selection criteria. Disagreements between reviewers will be resolved through discussion or by consulting a third reviewer. Finally, the study that meets the inclusion criteria will be included in the systematic literature review. | |
|  | Quality assessment: | The quality of the included studies will be assessed using the Newcastle-Ottawa Scale (NOS) for observational studies. The NOS consists of eight items that evaluate the quality of the study design and execution, and assigns a score of 0-9, with higher scores indicating higher quality. Two reviewers will independently assess the quality of the studies, and any discrepancies will be resolved through discussion. | |
|  | Data extraction | A data extraction table or summary will be prepared in Microsoft Word to extract important findings. This includes the type, nature, and design of the study, the study design, the main findings, and other relevant notes. The extracted data will then be analyzed to identify common trends and patterns among the studies. | |
|  | Data synthesis | A summary table will be prepared to describe the findings in each study. Then, a systematic literature review will be written by dividing the relevant findings into different themes and subthemes. Finally, a critical synthesis of the findings will be presented in order to draw conclusions from the systematic review. | |
|  | Ethical considerations: | This review will not involve any primary data collection and therefore does not raise any ethical concerns. | |
|  | Limitation | The review will be limited to studies published in English, which may exclude relevant studies published in other languages.  The review will be limited to studies published between January 2020 and the current date and may not capture the full impact of the COVID-19 pandemic on NCD patients in sub-Saharan African countries.  The review will not include a meta-analysis, and therefore will not be able to combine the results of the included studies in a quantitative manner. | |
