# Supplementary material for: The impact of COVID-19 on non-communicable disease patients in sub-Saharan African countries: A systematic review
Source: PLoS One. 2024 Jun 21;19(6):e0293376. doi: 10.1371/journal.pone.0293376 (PMC11192341; doi:10.1371/journal.pone.0293376)
Supplement: S2 File — (DOCX) [file pone.0293376.s004.docx]

## Systematic search strategy

Review question: What are the impacts of COVID-19 on NCDs patinates in sub-Saharan African countries?

The review question is divided into 3 concepts

Concept 1: Noncommunicable Diseases

Concept 2: COVID-19

Concept 3: Sub-saharan African Countries

Main terms and key words

Concept 1: Noncommunicable Diseases

Medline: (MM "Noncommunicable Diseases") OR (MM "Hypertension") OR (MM "Diabetes Mellitus") OR (MM "Pulmonary Disease, Chronic Obstructive")

*EMBASE: 'non communicable disease'/exp OR 'chronic disease'/exp OR 'hypertension'/exp OR 'diabetes mellitus'/exp OR 'chronic obstructive lung disease'/exp

*CINAHL: (MM "Noncommunicable Diseases") OR (MM "Hypertension") OR (MM "Diabetes Mellitus") OR (MM "Pulmonary Disease, Chronic Obstructive")

Keywords: ti OR ab (('non communicable*' OR 'non communicable*' OR noncommunicable*) NEAR/2 (illness* OR diseas* OR disorder* OR chronic)) OR asthma* OR cancer* OR neoplasm* OR copd* OR (('hypertensive' OR 'hypertension') NEAR/2 (acute OR arterial OR 'blood pressure high' OR 'cardiovascular' OR 'controlled' OR 'endocrine' OR 'high' OR 'disease' OR 'effect' OR 'response' OR 'neurogenic' OR 'preexistent' OR 'secondary' OR 'systemic')) OR 'high blood pressure' OR 'htn' OR 'hypertension' OR 'diabetes' OR 'diabetic' OR 'dm' OR ('chronic obstructi*' NEAR/2 'airflow') OR 'airway' OR 'bronchopulmonary disease' OR 'lung disorder' OR 'pulmonary disease' OR 'pulmonary disorder' OR 'respiratory disease' OR 'copd' OR 'lung disease' OR 'obstructive lung disease' OR 'chronic pulmonary disease' OR 'chronic obstructive pulmonary disorder'

*Keywords: AB OR TI (‘’non communicable*’’ OR non-communicable* OR noncommunicable*) NEAR/2 (illness* OR diseas* OR disorder* OR chronic) OR asthma* OR cancer* OR neoplasm* OR COPD* OR ((‘‘hypertensive’‘ OR ‘‘hypertension’‘) NEAR/2 (acute OR arterial OR ‘‘blood pressure high’‘ OR ‘‘cardiovascular’‘ OR ‘‘controlled’‘ OR ‘‘endocrine’‘ OR ‘‘high’‘ OR ‘‘disease’‘ OR ‘‘effect’‘ OR ‘‘response’‘ OR ‘‘neurogenic’‘ OR ‘‘preexistent’‘ OR ‘‘secondary’‘ OR ‘‘systemic’‘)) OR ‘‘high blood pressure’‘ OR ‘‘htn’‘ OR ‘‘hypertension’‘ OR ‘’diabetes’’ OR ‘’diabetic’’ OR (‘‘chronic obstructi*’‘ NEAR/2 ‘‘airflow’‘) OR ‘‘airway’‘ OR ‘‘bronchopulmonary disease’‘ OR ‘‘lung disorder’‘ OR ‘‘pulmonary disease’‘ OR ‘‘pulmonary disorder’‘ OR ‘‘respiratory disease’‘ OR ‘‘copd’‘ OR ‘‘lung disease’‘ OR ‘‘obstructive lung disease’‘ OR ‘‘chronic pulmonary disease’‘ OR ‘‘chronic obstructive pulmonary disorder’‘

Concept 2: COVID-19

*Medline (MH "sars-cov-2")

EMBASE: 'coronavirus disease 2019'/exp OR 'sars-cov-2 vaccine'/exp

*CINAHL: (MH "COVID-19") OR (MH "SARS-CoV-2") OR (MH "COVID-19 Pandemic")

Web of Science: no index search run on topic

EMBASE Keywords ti OR ab: ‘2019 Novel Coronavirus’ OR 2019-nCoV OR COVID OR ‘COVID 19’ OR COVID19 OR covid-19 OR ‘Coronavirus disease 2019’ OR ‘Coronavirus disease-2019’ OR ‘SARS Coronavirus 2 Infection*’ OR ‘SARS-CoV-2019’ OR 2019-nCoV OR ‘2019 nCoV’ OR ‘wuhan flu’ OR ‘2019 novel coronavirus’ OR ‘nCoV 2019’ OR ‘novel coronavirus 2019’ OR ‘Wuhan coronavirus*’ OR ‘novel coronavirus pneumonia’ OR ‘2019 novel coronavirus’ OR ‘coronavirus infection 2019’ OR ‘new coronavirus pneumonia’ OR ‘coronavirus infected pneumonia’ OR ‘long covid*’

*EBSCO Keywords: “2019 Novel Coronavirus” OR 2019-nCoV OR COVID OR “COVID 19” OR COVID19 OR covid-19 OR “Coronavirus disease 2019” OR ”Coronavirus disease-2019” OR “SARS Coronavirus 2 Infection*” OR “SARS-CoV-2019” OR 2019-nCoV OR “2019 nCoV” OR “wuhan flu” OR “2019 novel coronavirus” OR “nCoV 2019” OR “novel coronavirus 2019” OR “Wuhan coronavirus*” OR “novel coronavirus pneumonia” OR “2019 novel coronavirus” OR “coronavirus infection 2019” OR “new coronavirus pneumonia” OR “coronavirus infected pneumonia” OR “long covid*”

Concept 3: Sub-saharan Countries

EMBASE: 'africa south of the sahara'/exp

*MEDLINE: (MH "Africa South of the Sahara")

*CINAHL: (MM "Africa South of the Sahara")

Keywords: ti OR ab: 'sub-sahara*' OR 'sub sahara*' OR 'subsahara*' OR 'africa south of the sahara*' OR 'angola*' OR 'benin*' OR 'botswana*' OR 'burkina faso*' OR 'burundi*' OR 'cameroon*' OR 'cape verde*' OR 'central africa*' OR 'central african republic*' OR 'chad*' OR 'comoros*' OR 'congo*' OR 'cote d ivoire*' OR 'democratic republic congo' OR 'djibouti*' OR 'equatorial guinea*' OR 'eritrea*' OR 'eswatini*' OR 'ethiopia*' OR 'gabon*' OR 'gambia*' OR 'ghana*' OR 'guinea*' OR 'guinea-bissau*' OR 'kenya*' OR 'lesotho*' OR 'liberia*' OR 'madagascar*' OR 'malawi*' OR 'mali*' OR 'mayotte*' OR 'mozambique*' OR 'namibia*' OR 'niger*' OR 'nigeria*' OR 'rwanda*' OR 'sahel' OR 'senegal' OR 'sierra leone*' OR 'somalia*' OR 'south africa*' OR 'south sudan*' OR 'sudan*' OR 'tanzania*' OR 'togo*' OR 'uganda*' OR 'zambia*' OR 'zimbabwe*'

*Keywords: TI OR AB ‘‘sub-sahara*’‘ OR ‘‘sub sahara*’‘ OR ‘‘subsahara*’‘ OR ‘‘africa south of the sahara*’‘ OR ‘‘angola*’‘ OR ‘‘benin*’‘ OR ‘‘botswana*’‘ OR ‘‘burkina faso*’‘ OR ‘‘burundi*’‘ OR ‘‘cameroon*’‘ OR ‘‘cape verde*’‘ OR ‘‘central africa*’‘ OR ‘‘central african republic*’‘ OR ‘‘chad*’‘ OR ‘‘comoros*’‘ OR ‘‘congo*’‘ OR ‘‘cote d ivoire*’‘ OR ‘‘democratic republic congo’‘ OR ‘‘djibouti*’‘ OR ‘‘equatorial guinea*’‘ OR ‘‘eritrea*’‘ OR ‘‘eswatini*’‘ OR ‘‘ethiopia*’‘ OR ‘‘gabon*’‘ OR ‘‘gambia*’‘ OR ‘‘ghana*’‘ OR ‘‘guinea*’‘ OR ‘‘guinea-bissau*’‘ OR ‘‘kenya*’‘ OR ‘‘lesotho*’‘ OR ‘‘liberia*’‘ OR ‘‘madagascar*’‘ OR ‘‘malawi*’‘ OR ‘‘mali*’‘ OR ‘‘mayotte*’‘ OR ‘‘mozambique*’‘ OR ‘‘namibia*’‘ OR ‘‘niger*’‘ OR ‘‘nigeria*’‘ OR ‘‘rwanda*’‘ OR ‘‘sahel’‘ OR ‘‘senegal’‘ OR ‘‘sierra leone*’‘ OR ‘‘somalia*’‘ OR ‘‘south africa*’‘ OR ‘‘south sudan*’‘ OR ‘‘sudan*’‘ OR ‘‘tanzania*’‘ OR ‘‘‘‘togo*’‘ OR ‘‘uganda*’‘ OR ‘‘zambia*’‘ OR ‘‘zimbabwe*’‘

## Search results Embase search applied as an example

| No. | Query | Results | Date |
| --- | --- | --- | --- |
| #11 | #10 AND 'human'/de AND [adult]/lim AND 'Article'/it | 2902 | 03-Nov-22 |
| #10 | #3 AND #6 AND #9 | 10662 | 03-Nov-22 |
| #9 | #7 OR #8 | 2257007 | 03-Nov-22 |
| #8 | (ti OR ab) AND 'sub-sahara*' OR 'sub sahara*' OR 'subsahara*' OR 'africa south of the sahara*' OR 'angola*' OR 'benin*' OR 'botswana*' OR 'burkina faso*' OR 'burundi*' OR 'cameroon*' OR 'cape verde*' OR 'central africa*' OR 'central african republic*' OR 'chad*' OR 'comoros*' OR 'congo*' OR 'cote d ivoire*' OR 'democratic republic congo' OR 'djibouti*' OR 'equatorial guinea*' OR 'eritrea*' OR 'eswatini*' OR 'ethiopia*' OR 'gabon*' OR 'gambia*' OR 'ghana*' OR 'guinea*' OR 'guinea-bissau*' OR 'kenya*' OR 'lesotho*' OR 'liberia*' OR 'madagascar*' OR 'malawi*' OR 'mali*' OR 'mayotte*' OR 'mozambique*' OR 'namibia*' OR 'niger*' OR 'nigeria*' OR 'rwanda*' OR 'sahel' OR 'senegal' OR 'sierra leone*' OR 'somalia*' OR 'south africa*' OR 'south sudan*' OR 'sudan*' OR 'tanzania*' OR 'togo*' OR 'uganda*' OR 'zambia*' OR 'zimbabwe*' | 2256997 | 03-Nov-22 |
| #7 | 'africa south of the sahara'/exp | 302883 | 03-Nov-22 |
| #6 | #4 OR #5 | 337995 | 03-Nov-22 |
| #5 | (ti OR ab) AND '2019 novel coronavirus' OR covid OR covid19 OR 'covid 19' OR 'coronavirus disease 2019' OR 'coronavirus disease-2019' OR 'sars coronavirus 2 infection*' OR 'sars-cov-2019' OR '2019 ncov' OR 'wuhan flu' OR 'ncov 2019' OR 'novel coronavirus 2019' OR 'wuhan coronavirus*' OR 'novel coronavirus pneumonia' OR '2019 novel coronavirus' OR 'coronavirus infection 2019' OR 'new coronavirus pneumonia' OR 'coronavirus infected pneumonia' OR 'long covid*' | 336245 | 03-Nov-22 |
| #4 | 'coronavirus disease 2019'/exp OR 'sars-cov-2 vaccine'/exp | 270695 | 03-Nov-22 |
| #3 | #1 OR #2 | 8543075 | 03-Nov-22 |
| #2 | (ti OR ab) AND (('non communicable*' OR 'non communicable*' OR noncommunicable*) NEAR/2 (illness* OR diseas* OR disorder* OR chronic)) OR asthma* OR cancer* OR neoplasm* OR copd* OR (('hypertensive' OR 'hypertension') NEAR/2 (acute OR arterial OR 'blood pressure high' OR 'cardiovascular' OR 'controlled' OR 'endocrine' OR 'high' OR 'disease' OR 'effect' OR 'response' OR 'neurogenic' OR 'preexistent' OR 'secondary' OR 'systemic')) OR 'high blood pressure' OR 'htn' OR 'hypertension' OR 'diabetes' OR 'diabetic' OR 'dm' OR ('chronic obstructi*' NEAR/2 'airflow') OR 'airway' OR 'bronchopulmonary disease' OR 'lung disorder' OR 'pulmonary disease' OR 'pulmonary disorder' OR 'respiratory disease' OR 'copd' OR 'lung disease' OR 'obstructive lung disease' OR 'chronic pulmonary disease' OR 'chronic obstructive pulmonary disorder' | 8335289 | 03-Nov-22 |
| #1 | 'non communicable disease'/exp OR 'chronic disease'/exp OR 'hypertension'/exp OR 'diabetes mellitus'/exp OR 'chronic obstructive lung disease'/exp | 2145123 | 03-Nov-22 |
