# Supplementary material for: The impact of COVID-19 on non-communicable disease patients in sub-Saharan African countries: A systematic review
Source: PLoS One. 2024 Jun 21;19(6):e0293376. doi: 10.1371/journal.pone.0293376 (PMC11192341; doi:10.1371/journal.pone.0293376)
Supplement: S3 File — (DOCX) [file pone.0293376.s005.docx]

Subject: Response to Required Modifications for PONE-D-23-31770R2

Dear PLOS ONE Staff,

I hope this email finds you well. Thank you for your valuable feedback on our manuscript titled "The impact of COVID-19 on non-communicable disease patients in sub-Saharan African countries: a systematic review." We appreciate the thorough review process and the opportunity to address the suggested modifications.

Below, I have outlined the necessary revisions as per your instructions:

1. Author List and Affiliations:

We have carefully reviewed the author list and affiliations on the title page of our manuscript. All the listed authors have confirmed their roles, and we have not made any corrections. It's worth noting that the author name in the manuscript is Muluken Basa, while in the online registration system, it appears as Muluken Bafa Basa. The corresponding author prefers to be addressed in this publication as Muluken Basa, consistent with the manuscript.

Regarding Author Contributions, Competing Interests, and Financial Disclosure, no changes were needed as the initial information accurately reflects the contributions and funding status for each author.

2. Supporting Information:

To address the discrepancy in the number of Supporting Information captions and files, we have added the missing supporting information (S1 File: Review protocol). Originally omitted due to its availability on PROSPERO with the provided ID and citation in our manuscript, we believe this addition addresses the concern.

4. Author Formatting Checklist:

We have carefully gone through the Author Formatting Checklist to confirm that our paper meets PLOS ONE's typesetting requirements for References, Tables, and Figures. The necessary adjustments have been made to adhere to the guidelines. In addition, we have checked and updated the references accordingly.

5. PACE Tool for Figures:

Unfortunately, we do not have image files to run through the PACE tool, as we only have one figure, the Prisma diagram, which is available in Word document form. This figure complies with technical requirements.

We believe that these revisions address all the concerns raised in your email. The revised manuscript, along with the tracked changes in the attached document (PONE-D-23-31770R2_FTC.docx), is ready for your review.

If you have any further questions or require additional information, please do not hesitate to contact us at basam@tcd.ie. We appreciate your continued support and look forward to the potential publication of our manuscript in PLOS ONE.

Thank you for your time and consideration.

Kind regards,

Muluken Basa


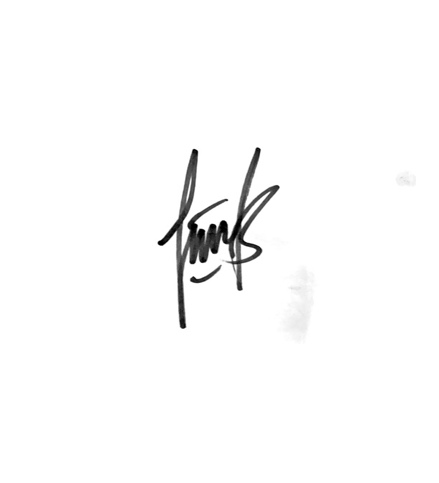


Corresponding author; PhD Candidate and researcher at School of Nursing and Midwifery, Trinity College Dublin.

24 D’Olier Street, TCD, Dublin 2

Email [basam@tcd.ie](mailto:basam@tcd.ie) or [mulerbafa@gmail.com](mailto:mulerbafa@gmail.com)

+353894748735/

Coauthors

Professor Catherine M. Comiskey, BA(Mod), MA, MSc, PhD, FTCD
Professor in Healthcare Modelling and Statistics, Academic Director of CHARM-EU, <https://www.charm-eu.eu/node/1>

Dr Jan De Vries, Associate Professor of Psychology and Subject Leader, School of Nursing and Midwifery, Trinity College Dublin

Dr David McDonagh, School of Nursing and Midwifery, Trinity College Dublin
